# Tension Pneumothorax During Surgery for Thoracic Spine Stabilization in Prone Position: A Case Report and Review of Literature

**DOI:** 10.1177/2324709614537233

**Published:** 2014-06-03

**Authors:** Demicha Rankin, Paul S. Mathew, Lakshmi N. Kurnutala, Suren Soghomonyan, Sergio D. Bergese

**Affiliations:** 1 Ohio State University Wexner Medical Center, Columbus, OH, USA

**Keywords:** tension pneumothorax, prone position, occult pneumothorax, thoracic spine surgery, thoracic trauma, intraoperative complications

## Abstract

The intraoperative progression of a simple or occult pneumothorax into a tension pneumothorax can be a devastating clinical scenario. Routine use of prophylactic thoracostomy prior to anesthesia and initiation of controlled ventilation in patients with simple or occult pneumothorax remains controversial. We report the case of a 75-year-old trauma patient with an insignificant pneumothorax on the right who developed an intraoperative tension pneumothorax on the left side while undergoing thoracic spine stabilization surgery in the prone position. Management of an intraoperative tension pneumothorax requires prompt recognition and treatment; however, the prone position presents an additional challenge of readily accessing the standard anatomic sites for pleural puncture and air drainage.

## Introduction

During general anesthesia and positive pressure ventilation, a simple pneumothorax can transition into a tension pneumothorax. Clinically, it can manifest as cardiovascular instability but the differential diagnoses for these symptoms can vary from circuit disconnection and endotracheal tube misplacement to allergic reaction. The likelihood of a tension pneumothorax increases when there are additional physical findings otherwise unexplained: elevated airway pressure, unilaterally decreased breath sounds, distended neck veins, or a deviated trachea. The development of an intraoperative tension pneumothorax during surgery in prone position can have devastating consequences, especially since the traditional sites for pleural puncture and tube insertion may not be easily accessible. Nevertheless, this complication warrants timely diagnosis and prompt management. A high index of suspicion and willingness to halt surgery for needle decompression or tube thoracostomy is required to prevent a potentially fatal outcome.

In this report, we present a trauma patient with a small pneumothorax on the right who developed a left-sided intraoperative tension pneumothorax while in prone position during surgery for thoracic spine stabilization.

The case report was prepared in accordance with the institutional review board regulations. A written informed consent was obtained from the patient to publish this report.

## Case Presentation

**Figure 1.**
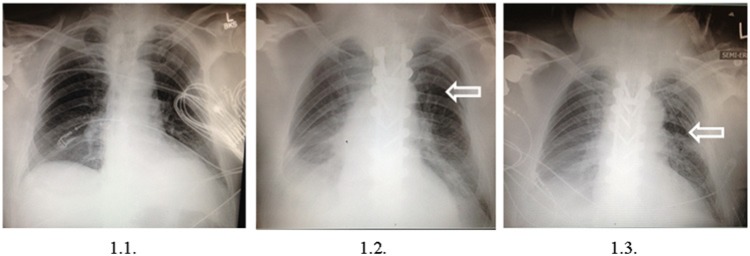
Results of the patient’s chest X-ray examinations. (1.1) Preoperative supine chest film. (1.2) Intraoperative supine film showing left pneumothorax. (1.3) Postoperative film showing left sided chest tube with improved lung markings.

A 75-year-old male with a history of ankylosing spondylitis was admitted to the hospital following a motor vehicle collision. Initially, he presented with a left lower extremity weakness and absent deep tendon reflexes. His past medical history was significant for arterial hypertension, hyperlipidemia, prostate cancer, and coronary arterial disease previously treated with coronary stent placement. A portable supine chest X-ray demonstrated low lung volumes with atelectasis of the left lung base, remote left-sided rib fractures, and an acute posterior left fifth rib fracture. No other abnormalities were reported (Figure 1-1). A computed tomography (CT) scan of the thorax showed complete transection of the thoracic spine at level of T_7-8_ with narrowing of the spinal canal, severe ankylosing spondylitis, a small hemothorax on the right, a small right apical pneumothorax, and an acute nondisplaced left fifth rib fracture. There was also evidence of remote trauma to the left second to fourth ribs. There were no major abnormalities in electrolytes and acid–base balance. General anesthesia was induced uneventfully, and surgery was attempted, but the procedure had to be aborted 2 hours into the procedure for refractory arterial hypotension. The patient was transferred to the intensive care unit intubated and sedated for further evaluation.

No cardiovascular factors could be identified to explain the intraoperative hypotensive episode, and a planned thoracic spine stabilization was arranged 48 hours after the first attempt. Of note, the patient had received positive pressure ventilation with low peak and plateau pressure levels while in the intensive care unit (ICU) and was placed on norepinephrine infusion to augment the spinal perfusion pressure.

After induction of anesthesia, the patient was positioned prone and the thoracic spine stabilization ensued as planned. After several hours and during the time of instrumentation, ventilation difficulties arose. A significant (>5%) decrease in end tidal CO_2_ level and an increase in peak airway pressure (>30 mm H_2_O) despite tracheobroncheal suctioning were recorded. Decreases in oxygen saturation were countered by increasing inspired oxygen concentration, which only resulted in 94% saturation indicating an increased intrapulmonary shunt via hypoventilated zones. Manual ventilation became increasingly challenging and the surgeon was asked to halt the procedure. Sterile drapes were applied to cover the open surgical incision, and the patient was returned to the supine position. An assessment for bilateral breath sounds was performed but proved inconclusive. A fiberoptic bronchoscopy demonstrated a small mucous plug but its removal did not improve the ventilation and gas exchange significantly. An arterial blood gas analysis at the time showed pH 7.31, PCO_2_ 48, and PO_2_ 99 mm Hg. An emergent supine chest X-ray revealed a *left-sided* pneumothorax (Figure 1.2). A chest thoracostomy tube placed into the left pleural space resulted in immediate improvement of lung compliance, hemodynamics, and restoration of lung markings on the early postoperative X-ray film (Figure 1.3). The patient was returned to the prone position with the thoracostomy tube in situ, and surgery was completed uneventfully.

## Discussion

The management of an occult or clinically insignificant pneumothorax in acute trauma patients has generated much debate among physicians.^1-3^ The term occult pneumothorax is used to reference those pneumothoraces that are not diagnosed on standard chest X-ray films but noted as incidental findings on subsequent chest CT scans.^1,4,5^ It is diagnosed in approximately 5% to 15% of hospitalized trauma populations and constitutes more than half of all the pneumothoraces encountered among trauma patients.^5,6^ An occult pneumothorax should be suspected if subcutaneous emphysema, pulmonary contusions, or rib fractures are present; however, only subcutaneous emphysema is independently predictive of occult pneumothorax (as cited in Ball et al^5^).

On the other hand, the small and clinically insignificant pneumothorax is a relatively common finding on chest X-rays of thoracic trauma patients. In most cases, the process is self-limited and resolves without causing any clinical consequences.

There appears to be a growing recognition and acceptance that in vast majority of cases an occult or a small pneumothorax can be safely treated without placing a thoracostomy tube in nonventilated or even mechanically ventilated patients.^2,3,7^ Conversely, Enderson et al^1^ prospectively randomized patients diagnosed with occult pneumothorax into observation and thoracostomy groups to follow the progression of the pneumothorax. In their study, 8 out of 21 patients in the observation group demonstrated progression of the occult pneumothorax, 3 of which developed a tension pneumothorax. The authors concluded that mechanically ventilated patients with an occult pneumothorax should be managed with a thoracostomy tube.^1^

In our patient, the tension pneumothorax was identified and managed in a timely manner. However, it raised important questions requiring clarification regarding the risks related to placing the acute trauma patients in prone position:

1. What criteria will warrant prophylactic placement of a thoracostomy tube?
2. Should unilateral or bilateral thoracostomy be performed?
3. Which is the preferred method for intraoperative emergent pleural space decompression if required?
4. Will an emergent decompression be feasible via the thoracic surgical incision?
5. What are the infection risks in the setting of exposed spinal hardware and an emergent breach of the sterile field?
6. What additional methods could be used to evaluate for a tension pneumothorax in a patient positioned prone on the operating table?

There is no clear evidence to drive the clinical decision, and the risk versus benefit must be examined closely. Thoracostomy has been reported to increase the overall mortality rate.^7^ However, considering the small number of patients in that report (22 patients completed the study) and low power of the study, no definitive conclusions can be made. We believe that in a subset of patients, prophylactic chest tube placement may help prevent serious life-threatening complications and ultimately decrease mortality.

In our patient, the clinical reasoning for initial observation was guided by the patient’s asymptomatic presentation. Hindsight allows for speculation that perhaps a prophylactic placement of a right thoracostomy tube prior to surgical intervention would be justified; however, this would have been of no value as the intraoperative tension pneumothorax developed on the left, and it is unlikely that a contralateral chest tube would be helpful. A supine chest X-ray was performed during the ICU interim stay, which did not reveal any new findings. Thus, the clinical suspicion was low in the asymptomatic patient. An upright chest X-ray was contraindicated given his unstable thoracic spine, and there was no indication to repeat a thoracic CT. Additionally, the patient received positive pressure ventilation for more than 24 hours prior to presenting to the operating theatre for the second time without progression to a tension pneumothorax in the right thorax.

We believe the propagating factors for development of the tension pneumothorax in this case were related to the displaced rib fracture on the left. It is possible that the rib fracture caused a pulmonary laceration creating a left sided pneumothorax during positioning of the first surgical attempt, perioperative management while in the ICU, or during the second surgical manipulation. The latter is especially probable given the downward displacement of the thoracic cage with instrumentation by the surgeon. This hypothesized pulmonary laceration could have created a pneumothorax that progressed into a tension pneumothorax under positive pressure ventilation.

Performing an emergent decompression in our patient was problematic because of the prone position, surgical drapes, and placement of arms parallel to the thorax. Returning the patient to the supine position was critical for timely management of tension pneumothorax and prevention of further deterioration.

Ultrasound has been shown to be more sensitive in the diagnosis of traumatic pneumothorax than the flat anteroposterior chest radiography.^8^ Using an animal model, Oveland et al^9^ showed that the thoracic ultrasound can be effectively used to monitor the progression of pneumothorax during mechanical ventilation. In cases when intraoperative development of tension pneumothorax is suspected, ultrasound imaging may be an easy and reliable diagnostic option, even in cases when the patient is in prone position. However, this assumption needs clinical validation.

The classical puncture site for tension pneumothorax is the second intercostal space in the midclavicular line; however, the fifth intercostal space on the anterior axillary line is an alternative site for emergent thoracostomy.^10^ We think that the utility of this latter approach in patients undergoing surgery in prone position deserves further evaluation.

## Conclusion

There is much controversy over the management of occult pneumothorax in patients receiving positive pressure ventilation. A multidisciplinary approach to such cases is required to develop an optimal treatment plan for patients who are at risk for developing a pneumothorax during surgical procedures. The patient’s position on the operating table and related difficulties that may prevent the immediate access for decompression should be taken into account.

Currently, there is no clear evidence that the benefit of placing a prophylactic chest-tube outweighs the risks related to thoracostomy. Clinical risk factors for our patient included the presence of a simple pneumothorax, an acute rib fracture, thoracic spine instrumentation/fixation, prone positioning, and positive pressure ventilation. We fully recognize that even in this case, a preoperative chest tube placement on the right side would have proved ineffective in the prevention of development of a tension pneumothorax on the left.

Successful management of such patients requires proper preoperative planning and a low threshold for intervention if needed. Intraoperative ultrasound examination should be considered as a valuable diagnostic method that can help confirm the diagnosis and localize the tension pneumothorax.^11-13^
